# Validation of BMP8A fibrosis score to identify patients with metabolic dysfunction-associated steatohepatitis with advanced liver fibrosis

**DOI:** 10.1186/s40364-025-00862-3

**Published:** 2025-11-19

**Authors:** Stephania C. Isaza, Carlos Ernesto Fernández-García, Diego Rojo, Paula Iruzubieta, Javier Ampuero, Rocío Aller, Raquel Vinuesa Campo, Laura Izquierdo-Sánchez, Esther Fuertes-Yebra, Patricia Marañón, Jesús M. Banales, Laura Pagés, Carolina Jiménez-González, Javier Rodríguez de Cía, Irene Olaizola, Judith Gómez-Camarero, Víctor Arroyo-Lopez, Manuel Romero-Gómez, Javier Crespo, Juan M. Pericàs, Carmelo García-Monzón, Águeda González-Rodríguez

**Affiliations:** 1 Metabolic Syndrome and Vascular Risk Laboratory, Hospital Universitario Santa Cristina, Instituto de Investigación Sanitaria del Hospital Universitario de La Princesa, Madrid, Spain; 2 Liver Unit, Vall d’Hebron University Hospital, Barcelona, Spain; 3 Liver Diseases Group, Vall d’Hebron Institute for Research, Barcelona, Spain; 4 Universitat Autònoma de Barcelona, Barcelona, Spain; 5 Gastroenterology and Hepatology Department, Marqués de Valdecilla University Hospital, Santander, Spain; 6 Clinical and Translational Research in Digestive Diseases, Valdecilla Research Institute (IDIVAL), Santander, Spain; 7 SeLiver Group, Instituto de Biomedicina de Sevilla/CSIC/Hospital Virgen del Rocío, Sevilla, Spain; 8 Centro de Investigación Biomédica en Red de Enfermedades Hepáticas y Digestivas (CIBEREHD), Madrid, Spain; 9 Servicio A Digestivo Hospital Clínico Universitario Valladolid, Universidad de Valladolid, Biocritic, Valladolid Spain; 10 Centro de Investigación Biomédica en Red de Enfermedades Infecciosas (CIBERINFEC), Madrid, Spain; 11 Fundación Burgos por la Investigación de la Salud, Burgos, Spain; 12 Department of Liver and Gastrointestinal Diseases, Biogipuzkoa Health Research Institute - Donostia University Hospital -, University of the Basque Country (UPV/EHU), Donostia-San Sebastian, Spain; 13 Ikerbasque, Basque Foundation for Science, Bilbao, Spain; 14 Department of Biochemistry and Genetics, School of Sciences, University of Navarra, Pamplona, Spain; 15 Servicio de Aparato Digestivo, Hospital Universitario de Burgos, Burgos, Spain; 16 Instituto de Investigaciones Biomédicas Sols-Morreale (Consejo Superior de Investigaciones Científicas-Universidad Autónoma de Madrid), Madrid, Spain; 17 Centro de Investigación Biomédica en Red de Diabetes y Enfermedades Metabólicas Asociadas (CIBERDEM), Madrid, Spain

**Keywords:** Advanced liver fibrosis, MASLD, MASH, BMP8A, BFS, Non-invasive diagnosis, Validation

## Abstract

Liver fibrosis represents the main risk factor not only for liver-related but also for overall mortality in metabolic dysfunction-associated steatotic liver disease (MASLD) patients, being metabolic dysfunction-associated steatohepatitis (MASH) its more severe clinical form. We recently developed a non-invasive algorithm termed BMP8A Fibrosis Score (BFS) which is able to identify MASH patients with advanced liver fibrosis. The aim of this study was to validate the BFS comparing its diagnostic accuracy with that of other scoring systems developed to assess liver fibrosis in MASH patients. Serum BMP8A was measured in 302 patients with biopsy-proven MASH: 171 with non- or mild fibrosis (F0-F2) and 131 with advanced fibrosis (F3-F4) recruited from seven university hospitals located in different cities in Spain. BFS, Fibrosis-4 (FIB-4) Index, NAFLD Fibrosis Score (NFS), Hepamet Fibrosis Score (HFS), and AST-to-Platelet Ratio Index (APRI) were calculated for each patient. The diagnostic accuracy of the scoring systems was determined according to the area under the receiver operating characteristic (AUROC) curve, sensitivity, specificity, positive (PPV) and negative (NPV) predictive values, and likelihood ratios (LR). BFS showed higher overall accuracy than the other liver fibrosis algorithms calculated in the study cohort, presenting an AUROC of 0.750 for predicting advanced liver fibrosis (F3-F4), and correctly classifying 70.9% of F3-F4 patients with a sensitivity of 58.0%, a specificity of 80.7%, a 71.5% NPV, a 69.7% PPV, a 3.0 LR+, and a 0.5 LR-; the other predictive scores correctly classified a lower percentage of these patients (63.6% for FIB-4 ≥ 2.67, 63.2% for HFS ≥ 0.47, 57.3% for APRI ≥ 1.5 and 56.9% for NFS ≥ 0.675). BFS eliminates the grey area as it uses a single cut-off value (0.46), which is its key advantage over the others, reducing the number of patients with undetermined results (43.4% for FIB-4, 39.1% APRI, 37.4% for HFS, and 24.1% NFS). In sum, BFS properly classified more patients with advanced liver fibrosis (F3-F4) than the other scoring systems, eliminating indeterminate results and improving risk stratification.

**Supplementary Information:**

The online version contains supplementary material available at 10.1186/s40364-025-00862-3.

To the editor

Metabolic dysfunction-associated steatotic liver disease (MASLD) is the world’s most common chronic liver disease (~ 38%), rising in diabetes and obesity [1]. Approximately, 25% of MASLD patients develop metabolic dysfunction-associated steatohepatitis (MASH), and about half of these are at risk of fibrosis progression. Fibrosis is the strongest predictor of long-term prognosis [2], so early detection is vital.

Liver biopsy remains the diagnostic gold standard, but it is invasive and impractical, highlighting the need for non-invasive biomarkers. Scores like Fibrosis-4 (FIB-4) Index [3], NAFLD Fibrosis Score (NFS) [4], Hepamet Fibrosis Score (HFS) [5], and AST-to-Platelet Ratio Index (APRI) [6] are widely used but limited by confounding factors and grey-zone results, leaving many patients unclassified [7].

We recently identified bone morphogenetic protein 8 A (BMP8A) as a potential biomarker for liver fibrosis since its serum concentration increases in fibrotic patients. Based on these findings, the BMP8A Fibrosis Score (BFS) was developed, integrating serum BMP8A, age, and platelet count, and was able to discriminate advanced liver fibrosis (F3-F4) with a good accuracy in MASH patients [8].

To validate BFS we conducted a study in independent cohorts of biopsy-proven MASLD patients (Supplemental Tables 1 and 2). Results showed that serum BMP8A levels were significantly higher in patients with advanced liver fibrosis (F3-F4) (339.6 ± 253.9 pg/mL) compared with those without or with mild fibrosis (F0-F2) (230.5 ± 142.3 pg/mL, *p* < 0.001) (Fig. 1A). BMP8A concentrations progressively increased across fibrosis stages, correlating with severity (Fig. 1B). Diagnostic performance was assessed using the area under the receiver operating characteristic (AUROC) curve analysis. BMP8A alone showed an AUROC of 0.669 (Fig. 1C), while BFS achieved 0.750 (Fig. 1D), though not outstanding, it outperformed FIB-4 (0.747), HFS (0.723), APRI (0.706), and NFS (0.650) (Fig. 1E).

**Fig. 1:**
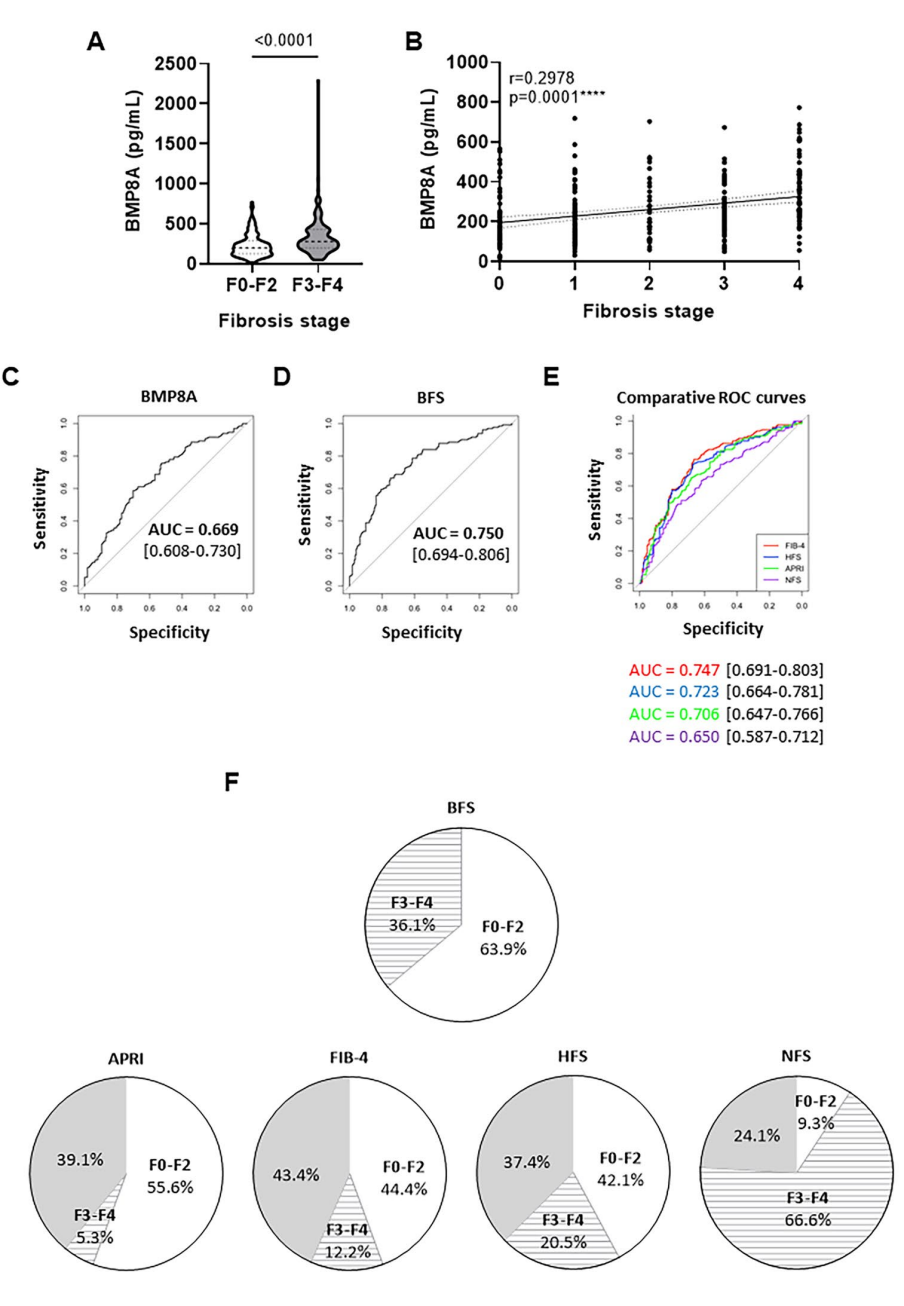
Validation of BFS as a non-invasive method for advanced fibrosis assessment. **(A)** Serum levels of BMP8A determined by ELISA. Data are expressed as pg/mL and presented as mean ± SD. **(B)** Correlation in the study population of matched serum BMP8A levels with fibrosis stage. **(C)** AUROC of BMP8A to predict advanced liver fibrosis (F3-F4). **(D)** AUROC of BFS to predict advanced liver fibrosis (F3-F4). **(E)** AUROCs of FIB-4, HFS, NFS and APRI to predict advanced liver fibrosis (F3-F4). **(F)** Graphical representation (%) of patients classified according to different predictive scores of advanced liver fibrosis (F3-F4). Study population: 302 MASH patients, 171 with non or mild liver fibrosis (F0-F2) and 131 with advanced fibrosis (F3-F4). BFS, BMP8A fibrosis score; FIB-4, Fibrosis 4 index; APRI, AST-to-platelet ratio index; NFS, NAFLD fibrosis score; HFS, Hepamet fibrosis score

Summing up Additional File 1, described in detail in Supplemental material, BFS ≥ 0.46, FIB-4 ≥ 2.67 and HFS ≥ 0.47 demonstrated better performance in confirming advanced liver fibrosis (F3-F4), whereas all algorithms (BFS < 0.46, FIB-4 < 1.30, HFS < 0.12, APRI < 0.5 and NFS<-1.447) showed ability to rule out disease. APRI ≥ 1.5 and NFS ≥ 0.675 showed very low diagnostic power for discrimination of advanced liver fibrosis (F3-F4) in this cohort.

Particularly, BFS demonstrated the highest overall accuracy, correctly classifying 70.9% of patients with advanced liver fibrosis (F3-F4), while the other predictive indices correctly classified a lower percentage of these patients. Notably, although sensitivity is lower (58%), maintaining a NPV of 71.5%, it should be noted that 63.9% of patients are classified as F0-F2 due to the absence of grey area, while FIB-4 < 1.30 and HFS < 0.12 achieved better sensitivity and NPV values at the expense of a significant number of indeterminate classifications (Fig. 1F). BFS also preserved a LR- of 0.5, supporting its capacity to effectively rule out advanced liver fibrosis (F3-F4). Additionally, BFS showed a specificity of 80.7% and a PPV of 69.7% to rule in advanced liver fibrosis (F3-F4). In fact, the PPV of BFS is higher than those of APRI ≥ 1.5, HFS ≥ 0.47 or NFS ≥ 0.675, while FIB-4 ≥ 2.67 showed the highest value. However, at this FIB-4 cut-off point, the LR- and sensitivity reflected the high number of false negatives and the inability to identify the majority of cases, which remain classified as indeterminate. Indeed, the highest cut-off points for FIB-4 and HFS included only 12.2% and 20.5% of patients, respectively, while the prevalence of patients with advanced liver fibrosis (F3-F4) in the validation cohort was 43.4%. In contrast, BFS included 36.1% of patients and performed better overall accuracy, showing better balance in discriminating advanced liver fibrosis (F3-F4) at a single cut-off point of 0.46 with LR + of 3.0 and LR- of 0.5.

These findings validate BFS as a non-invasive method for advanced liver fibrosis (F3-F4) assessment in MASLD. The study also highlights the broader context of non-invasive fibrosis evaluation. Ultrasound elastography and specialized serum biomarkers have emerged as alternatives, with meta-analyses supporting FIB-4 and NFS as effective tools [9]. However, their performance is hindered by indeterminate results. By contrast, BFS provides a definitive classification using one cut-off, making it particularly useful in clinical decision-making and clinical trial settings.

One limitation is that BFS requires measurement of serum BMP8A via ELISA, in addition to age and platelet count, making it more complex and costly than simpler scores derived from routine clinical data. This drawback parallels other specialized biomarkers [10–12], such as MACK3 [10], which improve accuracy but require additional assays, sometimes not available in commercial laboratories, as is the case with BFS. Nonetheless, BFS could be particularly valuable in pharmaceutical research and in reducing reliance on liver biopsy for fibrosis assessment. The multicenter design of this study, involving seven hospitals, strengthens the generalizability of the findings. However, further validation in independent cohorts and inter-laboratory reproducibility studies are needed.

BFS is a promising non-invasive biomarker for diagnosing advanced liver fibrosis (F3-F4) in MASLD. It correctly classifies more patients with advanced liver fibrosis (F3-F4) than standard scoring systems, eliminates the grey zone, and might become a valuable tool for clinical practice and research, potentially reducing the need for invasive liver biopsies.

## Supplementary Information

Below is the link to the electronic supplementary material.

Supplementary Material 1: Additional file 1. Comparison of the diagnostic performance of BFS and other commonly used algorithms to detect high risk of advanced liver fibrosis (F3-F4). BFS, BMP8A fibrosis score; FIB-4, Fibrosis 4 index; APRI, AST-to-platelet ratio index; NFS, NAFLD fibrosis score; HFS, Hepamet fibrosis score; %, number of patients; SN, Sensitivity; SP, Specificity; PPV, Positive predictive value; NPV, Negative predictive value; LR+, Positive likelihood ratio; LR-, Negative likelihood ratio.

Supplementary Material 2

Supplementary Material 3

Supplementary Material 4

## Data Availability

Authors declared that all and the other data supporting the findings of this study are available within the paper. The raw data that support the findings of this study are available from the corresponding author upon reasonable request.
