# Supplementary material for: Validation of BMP8A fibrosis score to identify patients with metabolic dysfunction-associated steatohepatitis with advanced liver fibrosis
Source: Biomark Res. 2025 Nov 19;13:149. doi: 10.1186/s40364-025-00862-3 (PMC12628818; doi:10.1186/s40364-025-00862-3)
Supplement: Supplementary file 1 — Supplementary Material 1: Additional file 1. Comparison of the diagnostic performance of BFS and other commonly used algorithms to detect high risk of advanced liver fibrosis (F3-F4). BFS, BMP8A fibrosis score; FIB-4, Fibrosis 4 index; APRI, AST-to-platelet ratio index; NFS, NAFLD fibrosis score; HFS, Hepamet fibrosis score; %, number of patients; SN, Sensitivity; SP, Specificity; PPV, Positive predictive value; NPV, Negative predictive value; LR+, Positive likelihood ratio; LR-, Negative likelihood ratio. [file 40364_2025_862_MOESM1_ESM.docx]

**Table 3. Comparison of the diagnostic performance of BFS and other commonly used algorithms**

**to detect high risk of advanced fibrosis (F3-F4) (MASH, n=302).**

| **Operating characteristics** | **BFS≥0.46** | **APRI<0.5** | **APRI≥1.5** | **FIB-4<1.30** | **FIB-4≥2.67** | **HFS<0.12** | **HFS≥0.47** | **NFS<-1.447** | **NFS≥0.675** |
| --- | --- | --- | --- | --- | --- | --- | --- | --- | --- |
| **Accuracy** | 70.9 | 66.6 | 57.3 | 69.2 | 63.6 | 65.6 | 63.2 | 47.3 | 56.9 |
| **%** | 36.1 | 55.6 | 5.3 | 44.4 | 12.2 | 42.1 | 20.5 | 9.3 | 66.6 |
| **SN** | 58.0 | 62.6 | 6.9 | 78.6 | 22.1 | 77.1 | 31.3 | 93.9 | 77.1 |
| **SP** | 80.7 | 69.6 | 95.9 | 62.0 | 95.3 | 56.7 | 87.7 | 11.7 | 41.5 |
| **PPV** | 69.7 | 61.2 | 56.2 | 61.3 | 78.4 | 57.7 | 66.1 | 44.9 | 50.2 |
| **NPV** | 71.5 | 70.8 | 57.3 | 79.1 | 61.5 | 76.4 | 62.5 | 71.4 | 70.3 |
| **LR+** | 3.0 | 2.1 | 1.7 | 2.1 | 4.7 | 1.8 | 2.5 | 1.1 | 1.3 |
| **LR-** | 0.5 | 0.5 | 1.0 | 0.3 | 0.8 | 0.4 | 0.8 | 0.5 | 0.5 |
