# Supplementary material for: Validation of BMP8A fibrosis score to identify patients with metabolic dysfunction-associated steatohepatitis with advanced liver fibrosis
Source: Biomark Res. 2025 Nov 19;13:149. doi: 10.1186/s40364-025-00862-3 (PMC12628818; doi:10.1186/s40364-025-00862-3)
Supplement: Supplementary file 2 — Supplementary Material 2 [file 40364_2025_862_MOESM2_ESM.docx]

**Supplementary Table 1. Characteristics of the study population**

| **Feature** | **Validation cohort**  **(n=302)** |
| --- | --- |
| Age (years) | 56.8 ± 10.1 |
| Gender |  |
| Women, n (%) | 159 (52.7) |
| Men, n (%) | 143 (47.3) |
| BMI (kg/m^2^) | 34.1 ± 6.2 |
| Glucose (mg/dL) | 122.4 ± 37.4 |
| Diabetes, n (%) | 163 (54.0) |
| Triglycerides (mg/dL) | 170.3 ± 79.1 |
| AST (IU/L) | 51.0 ± 65.5 |
| ALT (IU/L) | 60.6 ± 41.8 |
| GGT (IU/L) | 105.9 ± 130.0 |
| Platelets (10^9^/L) | 226.4 ± 65.8 |
| BMP8A (pg/mL) | 277.8 ± 205.4 |
| Fibrosis stage |  |
| F0-F2, n (%) | 171 (56.6) |
| F3-F4, n (%) | 131 (43.4) |

Data are shown as mean ± SD or as number of cases (%).

*BMI, body mass index; AST, aspartate aminotransferase; ALT, alanine aminotransferase; GGT, gamma-glutamyltransferase.*
