# Supplementary material for: Validation of BMP8A fibrosis score to identify patients with metabolic dysfunction-associated steatohepatitis with advanced liver fibrosis
Source: Biomark Res. 2025 Nov 19;13:149. doi: 10.1186/s40364-025-00862-3 (PMC12628818; doi:10.1186/s40364-025-00862-3)
Supplement: Supplementary file 3 — Supplementary Material 3 [file 40364_2025_862_MOESM3_ESM.docx]

**Supplementary Table 2. Characteristics of all patients based on the fibrosis stage**

| **Feature** | **F0-F2**  **(n=171)** | **F3-F4**  **(n=131)** |
| --- | --- | --- |
| Age (years) | 54.2 ± 10.5 | 60.2 ± 8.5*** |
| Gender |  |  |
| Women, n (%) | 92 (53.8) | 67 (51.1) |
| Men, n (%) | 79 (46.2) | 64 (48.9) |
| BMI (kg/m^2^) | 35.0 ± 6.7 | 32.8 ± 5.2** |
| Glucose (mg/dL) | 116.9 ± 33.2 | 129.5 ± 41.3** |
| Diabetes (%) | 78 (45.6) | 85 (64.9)*** |
| Triglycerides (mg/dL) | 166.9 ± 74.7 | 174.7 ± 84.7 |
| AST (IU/L) | 45.4 ± 71.6 | 58.4 ± 56.0*** |
| ALT (IU/L) | 56.7 ± 44.1 | 65.7 ± 38.2** |
| GGT (IU/L) | 81.8 ± 85.8 | 137.1 ± 166.5*** |
| Platelets (10^9^/L) | 246.1 ± 60.6 | 200.7 ± 63.8*** |
| BMP8A (pg/mL) | 230.5 ± 142.3 | 339.6 ± 253.9*** |

Data are shown as mean ± SD or as number of cases (%). **p<0.01; ***p<0.001.

*BMI, body mass index; AST, aspartate aminotransferase; ALT, alanine aminotransferase; GGT, gamma-glutamyltransferase.*
