# Supplementary material for: Validation of BMP8A fibrosis score to identify patients with metabolic dysfunction-associated steatohepatitis with advanced liver fibrosis
Source: Biomark Res. 2025 Nov 19;13:149. doi: 10.1186/s40364-025-00862-3 (PMC12628818; doi:10.1186/s40364-025-00862-3)
Supplement: Supplementary file 4 — Supplementary Material 4 [file 40364_2025_862_MOESM4_ESM.docx]

**SUPPLEMENTAL MATERIAL**

**MATERIAL AND METHODS**

***Study population***

This multicenter study included 302 patients with biopsy-proven MASH, of them 171 showed non- or mild fibrosis (F0-F2) and 131 displayed advanced fibrosis stages (F3-F4), extracted from the Spanish HEPAmet Registry. All patients met the inclusion criteria that included: age between 18 and 75 years old, less than 20 gr per day of alcohol consumption, absence of treatment with any potentially hepatotoxic drug, no analytical evidence of iron overload and seronegativity for HIV and hepatitis B and C infection. The diagnosis of MASH was based on histological criteria. All liver biopsies were assessed by locally experienced hepato-pathologists.

This study was performed in agreement with the Declaration of Helsinki, and with local and national laws. The Hospital Universitario de La Princesa Clinical Research Ethics Committee approved the study procedures (report reference, CEIm report 20/23), and all participants signed an informed written consent before inclusion in the study, providing permission for their medical data to be anonymously used for research.

Clinical and biochemical characteristics of each patient were routinely determined by central laboratories. Blood-based fibrosis scores such as FIB-4, APRI, NFS and HFS were calculated in the study population as previously described (3–6).

Detailed description of the study cohort is provided in Supplemental Table 1 and characteristics of all patients based on the fibrosis stage are shown in Supplemental Table 2.

***Determination of serum BMP8A levels***

Human BMP8A ELISA kit (CSB-EL002745HU, Cusabio Technology LLC, Hubei, China) was used to determine BMP8A content in serum samples, following manufacturer’s indications. Absorbance from samples was interpolated to a standard curve using a four-parameter logistic (4-PL) equation.

***Statistical analysis.***

Kolmogorov-Smirnov test was applied to evaluate if the variables were adjusted or not to a normal distribution. Qualitative variables are presented as relative frequencies, and data between groups compared with Pearson’s Chi-squared or Fisher’s exact test as appropriate. Quantitative variables are expressed as measures of central tendency (mean) and dispersion (standard deviation -SD-). Data between groups were compared with Student´s *t* test for variables following a normal distribution and Mann-Whitney U test for continuous variables following a non-parametric distribution. In order to assess diagnostic accuracy of BFS and distinct scoring systems for liver fibrosis, receiver operating characteristics (ROC) curves and area under the ROC curve (AUROC) were carried out. All statistical analyses were performed using the GraphPad Prism 6.0 software (GraphPad Software Inc., San Diego, CA, USA) and the R software (version 4.4.1), with two-sided tests, with a p value of <0.05 considered as statistically significant.

**RESULTS**

***Performance of non-invasive tests for advanced liver fibrosis in the study population.***

BFS showed the highest AUROC, followed by that of FIB-4 (0.747; CI 95% 0.691-0.803), followed by HFS (0.723; 95% CI: 0.664-0.781), APRI (0.706; 95% CI: 0.647-0.766), and NFS (0.650; 95% CI: 0.587-0.712). The comparison of the AUROCs only revealed significant differences in discriminatory ability between BFS, FIB-4 and HFS compared with NFS (p=0.003, 0.002 and 0.021, respectively), and between FIB-4 and APRI (p=0.0042).

The predictive tests evaluated showed significant variability in their ability to detect advanced fibrosis in MASH (Additional file 1).

BFS with a threshold of ≥0.46, previously established in the estimation cohort (6), showed a sensitivity of 58.0% and specificity of 80.7%. The positive predictive value (PPV) was 69.7% and negative predictive value (NPV) was 71.5%, while the diagnostic accuracy was 70.9%. The positive likelihood ratio (LR+) was 3.0, indicating that a positive BFS result increases the probability of advanced fibrosis. The negative likelihood ratio (LR-) was 0.5, suggesting that a negative result decreases the probability of advanced fibrosis.

For APRI<0.5, sensitivity was 62.6% and specificity 69.6%. PPV was 61.2%, while NPV reached 70.8%, with a diagnostic accuracy of 66.6%. The LR+ was 2.1 and LR- was 0.5, indicating that a negative result moderately decreases the probability of disease. For APRI≥1.5, the sensitivity was 6.9% and specificity was 95.9%. In this case, PPV was 56.2% and NPV was 57.3%, with a diagnostic accuracy of 57.3%. The LR+ was 1.7, and the LR- was 1.0, suggesting a very limited ability to confirm disease.

For FIB-4<1.30, sensitivity was 78.6% and specificity was 62.0%. PPV was 61.3% and NPV was 79.1%, with a diagnostic accuracy of 69.2%. The LR+ was 2.1 and LR- was 0.3, indicating that this score is useful for ruling out advanced fibrosis. In contrast, for FIB-4≥2.67, sensitivity decreased to 22.1%, but specificity increased to 95.3%. The PPV in this case was 78.4% and NPV 61.5%, with a diagnostic accuracy of 63.6%. The LR+ was 4.7, indicating that a positive result at this threshold is useful for confirming advanced fibrosis, although the LR- was 0.8, offering a limited capacity to exclude disease.

For HFS<0.12, sensitivity was 77.1% and specificity was 56.7%. PPV was 57.7%, while NPV reached 76.4%, with a diagnostic accuracy of 65.6%. The LR+ was 1.8, while the LR- of 0.4 suggested that a negative result has a modest ability to rule out advanced fibrosis. For HFS≥0.47, sensitivity decreased to 31.3%, while specificity was 87.7%. In this case, PPV was 66.1% and NPV was 62.5%, with a diagnostic accuracy of 63.2%. The LR+ was 2.5 and the LR- was 0.8, indicating that this score has a good capacity to positively discriminate advanced fibrosis, but limited capacity to exclude it.

For NFS<-1.447, the sensitivity was 93.9% and the specificity was 11.7%. PPV was 44.9% and NPV was 71.4%, with a diagnostic accuracy of 47.3%. The LR+ was 1.1 and LR- was 0.5, suggesting that this threshold slightly increased the ability to exclude advanced fibrosis. In contrast, for NFS≥0.675, sensitivity was 77.1% and specificity was 41.5%. PPV was 50.2% and NPV was 70.3%, with a diagnostic accuracy of 56.9%. The LR+ was 1.3 and the LR- was 0.5, suggesting that this score, at this threshold, provides little relevant diagnostic information.
